# Application of convolutional neural network on early human embryo segmentation during in vitro fertilization

**DOI:** 10.1111/jcmm.16288

**Published:** 2021-01-24

**Authors:** Mingpeng Zhao, Murong Xu, Hanhui Li, Odai Alqawasmeh, Jacqueline Pui Wah Chung, Tin Chiu Li, Tin‐Lap Lee, Patrick Ming‐Kuen Tang, David Yiu Leung Chan

**Affiliations:** ^1^ Assisted Reproductive Technology Unit Department of Obstetrics and Gynaecology Faculty of Medicine The Chinese University of Hong Kong Hong Kong China; ^2^ School of Biomedical Sciences Faculty of Medicine The Chinese University of Hong Kong Hong Kong China; ^3^ School of Computer Science and Information Security Guilin University of Electronic and Technology Guilin China; ^4^ Department of Anatomical and Cellular Pathology State Key Laboratory of Translational Oncology Prince of Wales Hospital The Chinese University of Hong Kong Hong Kong China

**Keywords:** convolutional neural network, cytoplasm, day‐one human embryo segmentation, pronucleus, time‐lapse imaging, zona pellucida

## Abstract

Selection of the best quality embryo is the key for a faithful implantation in *in vitro* fertilization (IVF) practice. However, the process of evaluating numerous images captured by time‐lapse imaging (TLI) system is time‐consuming and some important features cannot be recognized by naked eyes. Convolutional neural network (CNN) is used in medical imaging yet in IVF. The study aims to apply CNN on day‐one human embryo TLI. We first presented CNN algorithm for day‐one human embryo segmentation on three distinct features: zona pellucida (ZP), cytoplasm and pronucleus (PN). We tested the CNN performance compared side‐by‐side with manual labelling by clinical embryologist, then measured the segmented day‐one human embryo parameters and compared them with literature reported values. The precisions of segmentation were that cytoplasm over 97%, PN over 84% and ZP around 80%. For the morphometrics data of cytoplasm, ZP and PN, the results were comparable with those reported in literatures, which showed high reproducibility and consistency. The CNN system provides fast and stable analytical outcome to improve work efficiency in IVF setting. To conclude, our CNN system is potential to be applied in practice for day‐one human embryo segmentation as a robust tool with high precision, reproducibility and speed.

## 1 INTRODUCTION

*In vitro* fertilization (IVF) has contributed to more than 8 million births since the first birth in 1978.^1^, ^2^ With four decades of efforts, IVF has developed into an available, efficient and safe assisted reproductive technology (ART) for infertile couples. After ovarian hyperstimulation, multiple embryos can be obtained while only one or two will be selected for embryo transfer. Thus, choosing embryos with highest developmental potential from the same batch of controlled hyperstimulated oocyte‐derived embryos is one of the most important tasks of IVF specialists.^3^ Up to date, the conventional morphology assessment is still the mainstream method for embryo selection either during the cleavage or blastocyst stage.^3^, ^4^, ^5^, ^6^, ^7^, ^8^ In contrast, the evaluation of a day‐one human embryo is generally considered to have little value except for fertilization check.^9^ Day‐one human embryo, as the first stage of an embryo begins at the fertilization however before the first embryo cleavage, plays an essential role in embryo development and thus the morphology assessment should attract more attention.

The important features of a day‐one human embryo include the zona pellucida (ZP), cytoplasm and pronucleus (PN, during a faithful fertilization). Studies have reported the associations between the embryo development and the three morphological features. Detecting the abnormal darkness, thickness and birefringence of ZP precisely may contribute to predict a successful hatching event that can lead to successful implantation.^10^ Cytoplasm occupies the largest portion of the day‐one human embryo, and the associations of the area size (at 2D level) and embryo development were reported.^11^ The conventional assessment of PN on the day‐one human embryo is mainly based on the number, size and location of PN. However, the results of the associations of PN locations and embryo quality remain controversial.^12^, ^13^, ^14^

To select embryos with high quality, time‐lapse imaging (TLI) technology has been introduced in IVF laboratories with morphokinetics scoring system.^15^ By using the in‐built microscope and camera, the embryos can be assessed *in situ* without taking them out of the incubator, thus giving undisturbed culture conditions.^16^ Moreover, images of the developing embryo are taken every five to ten minutes. As a result, a sequence of images of embryo development is generated as a time‐lapse video that can provide more information than the traditional method. However, analysing the time‐lapse video is time‐consuming, and some short appearing features cannot be properly captured or recognized by human naked eyes.

Convolutional neural network (CNN) is a deep learning architecture mimicking the natural mammalian visual perception system.^17^ It can obtain effective representations of the original image, which makes it possible to recognize visual patterns directly from raw pixels with little‐to‐none pre‐processing.^18^ At present, it has been applied in medical image segmentation, lesion detection, image classification and retrieval.^19^ For instance, CNN was used to segment computer tomography images of liver, head and neck, which were of assistance in radiotherapy treatment, post‐operative follow‐up, organs‐at‐risk detection and atherosclerosis perdiction.^20^, ^21^, ^22^

In the field of reproductive medicine, TLI generates massive image data, which requires embryologists to evaluate each image in the sequence to select a good‐quality embryo.^16^ The assistance of TLI gets the evaluation into trouble due to the time‐consuming process and minor changes that might be overlooked. CNN may be a potential tool to solve this bottleneck. The study aims to evaluate whether CNN for day‐one human embryo morphokinetic features segmentation could be applied in practice as a robust tool with high precision and reproducibility. We also examined the segmented values from the morphokinetic parameters of day‐one human embryo cytoplasm, ZP and PN, and compared the values with those in other studies to further confirm the precision of the segmentation algorithm.

## 2 MATERIALS AND METHODS

### 2.1 Ethics approval

The inform consent was obtained from all patients. This study was approved by the Joint Chinese University of Hong Kong—New Territories East Cluster Clinical Research Ethics Committee (CREC No: 2017.580).

### 2.2 Human time‐lapse images

#### 2.2.1 Fertilized oocyte for TLI

The studies were conducted from 2017.07.01 to 2019.12.31, in ART Unit, Department of Obstetrics and Gynaecology, Faculty of Medicine, The Chinese University of Hong Kong.

We set the inclusion and exclusion criteria in this study.

Inclusion criteria:

1. Consecutive women underwent IVF treatment.
2. Patients planned to use a time‐lapse incubator for embryo culture.

Exclusion criteria:

Day‐one human embryos with blur imaging, large obstructions on embryo area, more than half embryo area blocked by the well or degeneration, transferred or cryopreserved before day 5 were defined as incorrectly segmented embryos. Patients had more than half of her embryos which were not able to be segmented correctly were excluded.

#### 2.2.2 Ovarian stimulation, retrieval and fertilization

In the process of ovarian stimulation and retrieval, we referred to the information provided in a published paper.^23^ Briefly, the long luteal gonadotropin‐releasing hormone (GnRH) agonist was used to down‐regulate the pituitary. Buserelin nasal spray (Suprecur; Hoechst, Hørsholm, Germany) was given to the cases for no less than 14 days from the midluteal phase of the preceding cycle. The concentrations of low serum luteinizing hormone and oestradiol (E_2_) were used for confirming the complete pituitary desensitization. Additionally, the ultrasound examination was used to exclude the functional ovarian cysts and ensure the thickness of endometrial (<5 mm). After achieving adequate down‐regulation, human menopausal gonadotropin (hMG) (Pergonal; Serono, Aubonne/Switzerland) or recombinant follicle‐stimulating hormone (FSH) (Gonad‐F; Serono; or Puregon; Organon, Skovlunde, Holland) was used to start the ovarian stimulation. The dose was decided in regard to the ages and the previous treatment responses. The presence of more than three mature follicles (>18 mm) was considered as adequate responses. The transvaginal oocyte retrieval was conducted about 36 hours later.

After retrievals, the fertilization of the embryos was conducted like that described in another published paper.^24^ Hyaluronidase (Vitrolife, Goteborg, Sweden) was used to remove the cumulus cells, followed by cultivation for more than 1 hour. Four to Six hours after the retrievals, insemination by IVF or intracytoplasmic sperm injection (ICSI) was conducted.

#### 2.2.3 TLI setting

The time‐lapse incubator used in this study for TLI was EmbryoScope^®^, with the interval of 10 minutes. The CO_2_ was set as 6.0%, and the temperature was maintained at 37.0°C. The one‐step culture medium G‐TL (Vitrolife, Switzerland) was used for routine embryo culture, which is the bicarbonate buffered medium containing human serum albumin and hyaluronan.

For the incubation of embryos, we used the EmbryoSlide^®^ (Vitrolife, Switzerland). The incubator can hold up six dishes. Each dish can culture twelve embryos at one time, and it has individually numbered wells inside. After filling in the medium in the wells, oil was quickly used to overlay the medium to avoid the evaporation. The process of both the medium preparation and oil overlay was maintained at a cold temperature to avoid the evaporation as well. The dishes were kept overnight to make them balanced and then the embryos were loaded. The diameter of EmbryoSlide^®^ (Vitrolife, Switzerland) is 250 μm. Therefore, the total area of the well was 49062.5 μm^2^. We have measured the number of pixels of the well of the culture dish in all the time‐lapse images. The number of pixels inside the well was 16077.98 ± 192.35. The relationship between a pixel and its actual size was 1 pixel = 0.3275 μm^2^.

### 2.3 CNN for day‐one embryo morphology segmentation on images of TLI

CNN usually consists of the convolution layer, pooling layer and fully connected layer. The convolutional layer detects and extracts the visual features of images.^25^ The feature maps generated from the convolutional layer are processed by the pooling layer, and these layers repeat several times.^26^, ^27^ At last, the information extracted is processed by the fully connected layer (Supporting Information 1‐I).^28^

The CNN system was set in an environment with ubuntu 20 Operation system, 1080 Ti graphics processing unite, i7‐8700 central processing unit (3.2 GHz) and 16G random access memory. Our method contained two types of neural networks: one was the generative adversarial network for enhancing images; the other one was the hierarchical fully convolutional network for segmenting enhanced images. The generative adversarial network denoised and highlighted the area of interest in the images, consequently reducing the difficulty for the following procedures. The hierarchical fully convolutional network constrained the spatial relations among the areas of interest during segmentation and hence increased the accuracy of segmentation.

Firstly, we fixed the images collected from TLI in a uniform scale and converted them into grey images. The original image was compressed into 512*512 pixels with the greyscale ranging from 0 to 255. The bilateral filter with different smoothing coefficients was used to smooth and denoise the input images. Next, we trained the generative adversarial network for enhancing images and the structure of the generative adversarial network here was cycleGAN trained by the Adam algorithm.^29^ Then, we used the enhanced images dataset gained to deliver the image segmentation training dataset. After training the hierarchical fully convolutional network, we conducted image segmentation using the dataset. In this network, the input was the enhanced images. At the same time, the output was each pixel’s probabilities of belonging to the background, cytoplasm, ZP and nucleus of the whole image.

### 2.4 Day‐one embryo morphometrics labelling

The structures of the embryos, namely ZP, cytoplasm and PN, in each image were labelled by two experienced embryologists.

We used solid colours (Red: #FF0000, Yellow: #FFFF00 and Blue: #0000FF) to label the edge of each structure (i.e. ZP, cytoplasm and PN). The pure colours were easy for the computer to recognize the input information. When labelling, we magnified an image and focused on the structure of interest. Then, we confirmed the edge of the structure and marked down the trajectory with pure colour. Once one embryologist had finished the labelling for one sample, another embryologist examined the labelled result by naked eyes.

### 2.5 Statistical analysis

#### 2.5.1 Cross‐validation for day‐one embryo static cytoplasm segmentation

The experimental design of rotating the images between the test and training followed the widely used statistical cross‐validation protocol.^30^ The purpose of cross‐validation is to lower the risk of overestimating or underestimating the true performance of the proposed system, which is achieved by out‐of‐sample testing. We used cross‐validation to train and test the performance of the proposed system. We divided the dataset into 5 parts randomly. One part was chosen as the test data and the others were chosen as the training data. Each part was designed as the test data once in 5 trials. Then, we trained five separate recognition systems using four out of the five subsets and performed validation of the fifth hold‐out subset (Supporting Information 1‐II).

#### 2.5.2 Intersection over Union (IoU)

The Intersection over Union was a statistic used for gauging the similarity and diversity of sample sets. The IoU (a, b) between the predicted area a and the ground truth b is defined as *IoU* (*a*, *b*) = |a ∩ b|/|a ∪ b| (Supporting Information 1‐III).

Then, the difference of IoUs between the training phase and test phase in each trial as well as between trials was analysed with the Student’s *t* test. The *P*‐value was set as 0.05 to reject the null hypothesis.

#### 2.5.3 Comparison of continuous data

For normally distributed continuous variables, the Student t test was used to compare the difference. For non‐normal distribution parameters, the Wilcoxon signed‐rank test was used. The comparison was two‐sided, and the *P*‐value of significance was set as <0.05.

#### 2.5.4 Augmented dickey‐fuller test (ADF)

The ADF test is a method for detecting unit roots in autoregressive and autoregressive moving average time series. The presence of a unit root indicates the time series was not stationary, but that differencing would reduce it to stationarity.^31^ The null hypothesis of it was that the data were non‐stationary. The *P*‐value was set as 0.05 to reject the null hypothesis.

The cut‐off for defining whether a parameter was stationary was set as 70%, which indicated for a parameter, if over 30% of the samples were accepted the stationary hypothesis of the ADF test, it would be considered as a stationary parameter.

#### 2.5.5 Shapiro‐Wilk test

The Shapiro‐Wilk test is a test of normality in frequentist statistics.^32^ The null hypothesis of this test was that the population is normally distributed. The *P*‐value was set as 0.05 to reject the null hypothesis.

## 3 RESULTS

### 3.1 Day‐one human embryo morphokinetic features segmentation in time‐lapse images

**FIGURE 1:**
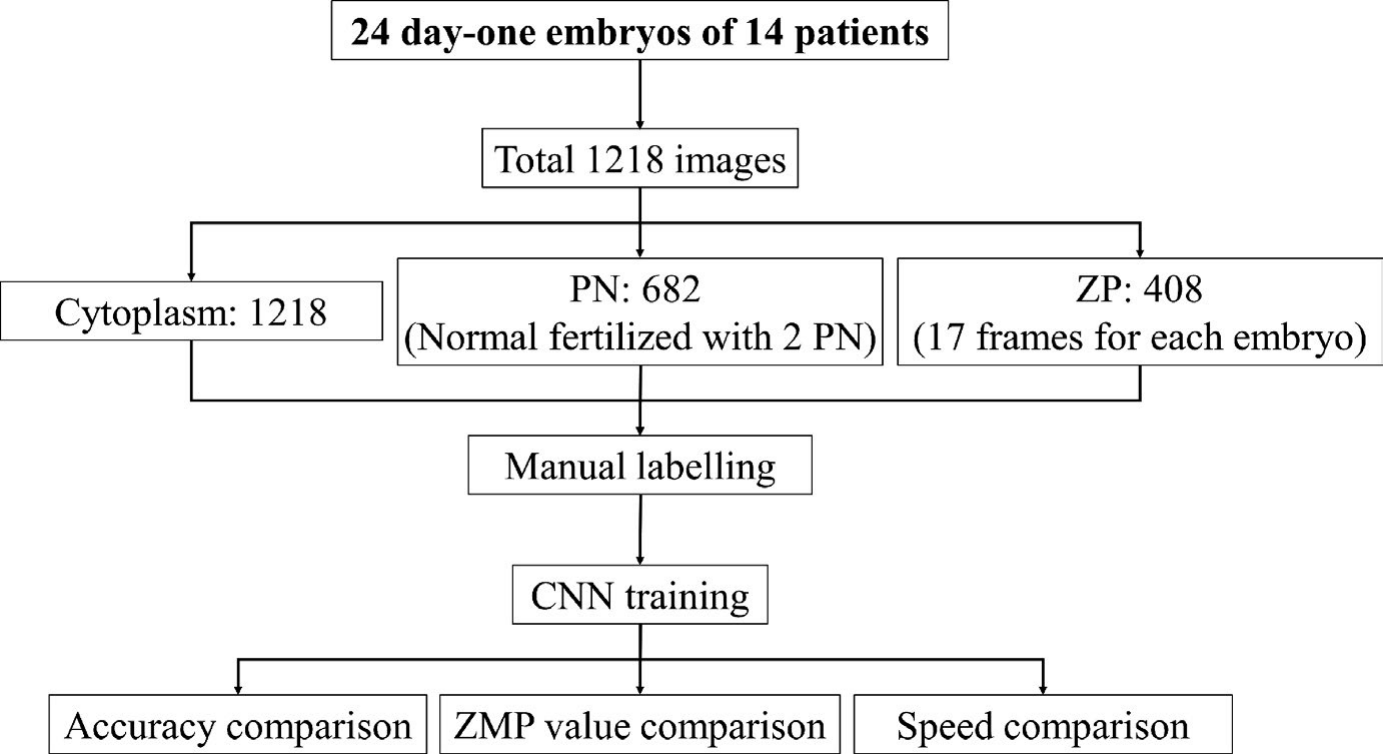
Flowchart of the CNN system. From 24 day‐one embryos of 14 patients, we obtained total 1218 images. Among the images, the cytoplasm, PN and ZP were labelled by experience embryologists. We trained the CNN system with the labelled image and compared its performance with experts. With the results, the accuracies, ZMP values and elapsed time of two methods were compared to validate the advantages of the CNN system

There were 1218 images obtained from 24 day‐one embryos of 14 patients. The demographic information of the patients is shown in the Table 1. We labelled 1218 for cytoplasm segmentation, 682 for PN recognition and 408 for ZP recognition. The differences of the numbers of labelled structures are that 1) the PN did not exist for the whole length of the embryos development and in this dataset, we can only label 682 frames of embryos; we only selected the images of the normal fertilized embryo with 2PN for training; 2) from the prior knowledge of embryology, the ZP would not change during the development of the embryo stage so that our embryologists labelled only 17 frames of each embryo, which was to reduce the workload of labelling. The flowchart of the CNN system is shown in the Figure 1.

**TABLE 1:** Demographic information of the patients

	Total (n = 14) (Median & IQR)
Insemination: IVF/ICSI	7/7
Age (year)	35 (34‐38)
E2 at trigger (pmol/L)	13491.5 (10694.75‐27180.0)
LH at trigger (IU/L)	2.55 (1.025‐3.17)
P4 at trigger (nmol/L)	2.625 (1.05‐3.725)
Gn dose total (mg)	1.5 (1.5‐1.75)
Stimulation period (day)	10.5 (10‐12)
Protocol: Antagonist (Flexible)/Antagonist pre‐treatment OC	9/5

Note

The method of insemination, age, level of different hormones at trigger, treated dose, stimulation period and treatment protocol was presented in the table to show the characteristics of the participants with median and interquartile.

Abbreviations: IQR, interquartile range; E2, estradiol; LH, luteinizing hormone; P4, progestrone; Gn, gonadotropin; IVF, in vitro fertilization; ICSI, Intracytoplasmic sperm injection; OC, oral contraceptive.

#### 3.1.1 Cytoplasm segmentation

**FIGURE 2:**
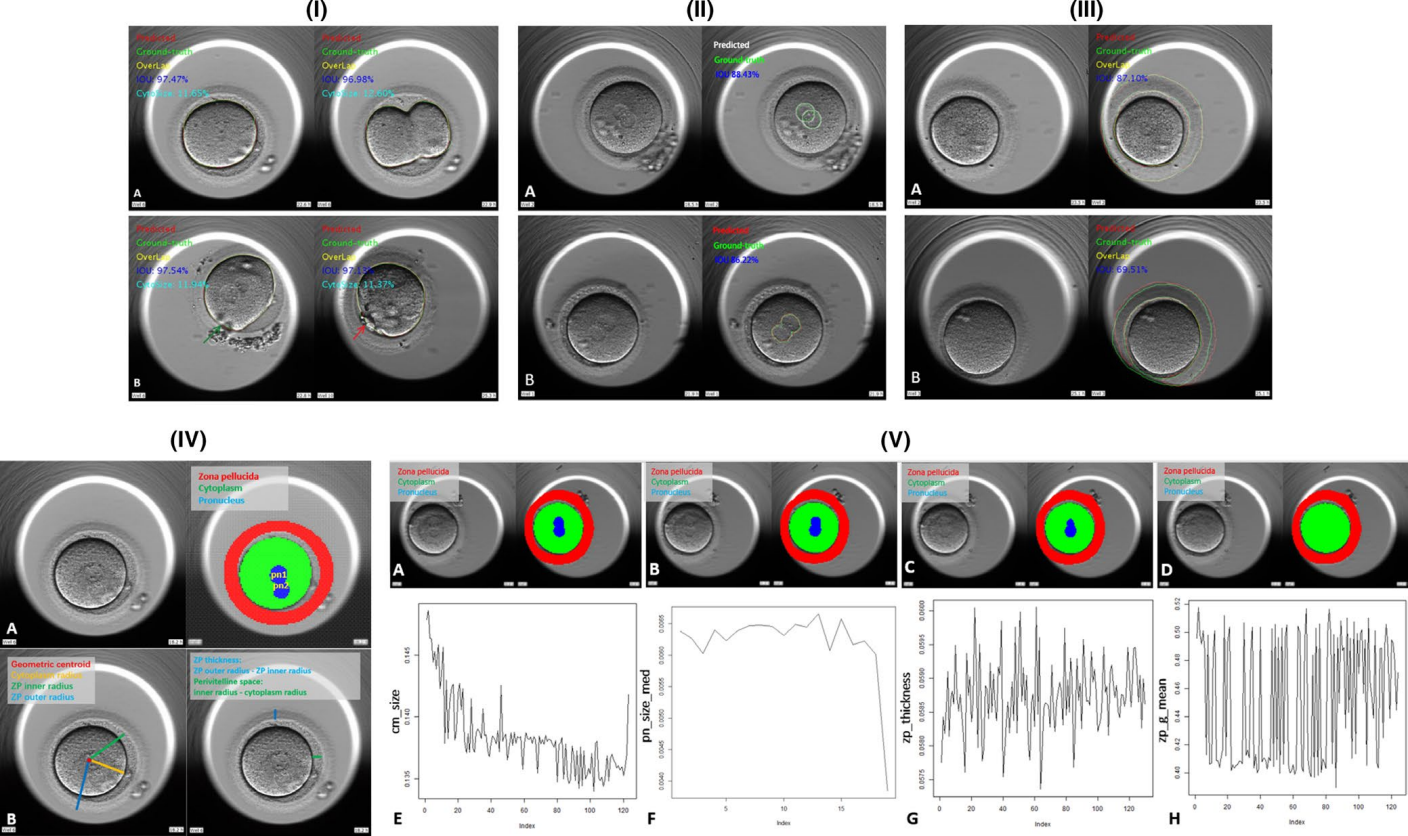
(I) Illustration of the segmentation of cytoplasm: Red circles represented the predicted area, while the green circle represented the labelled area (ground truth). The yellow circle represented the overlap of labelled and ground truth. IoU and cytoplasm size (CytoSize) were shown in the left upper corner. In Figure 2‐I‐A, two images came from the same zygote. The one on the left was captured before cleavage while the one on the right is cleaving, which was an irregular shape. The performances of segmentation on these two images were over 95%. In Figure 2‐I‐B, on the left image, cumulus cells (green arrow) blocked a part of the cytoplasm edge while on the right image; a big PB blocked the edge of another cytoplasm. Nevertheless, the IoU in both images was over 97% indicating the noise robust of the proposed system. (II) Illustration of the segmentation of PN: Figure 2‐II‐A showed the segmentation of respective PN, and green circles represented the labelled area while the white circles represent the segmented area. We used white colour to distinguish the type of segmentation with Figure 2‐II‐B. The average IoU of Figure 2‐II‐A was 88.43%. Figure 2‐II‐B showed the segmentation of fusion PN. The boundaries between PN were blurred so the labelled area was considered as the whole green circle and the segmented area was the whole red circle, correspondingly. The IoU of Figure 2‐II‐B was 86.22%. (III) Illustration of the segmentation of ZP: Figure 2‐III‐A showed the example of high precision (87.10%) segmentation of ZP. The green circle represented the labelled area, while the red circle represented the segmented area. Only a small part of the ZP was blocked by the edge of the well (from seven o’clock position to nine o’clock position). Figure III‐B showed the example of low precision (69.51%) segmentation of ZP. More than one‐fourth ZP was blocked by the edge of the well (from five o’clock position to nine o’clock position). Moreover, the ZP in seven o’clock position was hardly seen, which was not available for labelling by the embryologists or precise segmentation by the proposed system. (IV) Illustration of the calculation of zygote morphokinetic parameters: Figure 2‐IV‐A left was an original image captured by time‐lapse incubator and Figure 2‐IV‐A right was the pixelated and segmented one. The pixels belonged to ZP were labelled in red; the pixels belonged to cytoplasm were labelled in green, and the pixels belonged to PN were labelled in blue. It was easy to notice that if a pixel belonged to one of the structures but not located at the edge, all its neighbours were the pixels in the same colour. To distinct the PN, we defined the one closer to the centroid of cytoplasm as pn1, and the other as pn2. Figure 2‐IV‐B left showed the centroid (red), cytoplasm radius (yellow), ZP inner radius (green) and outer radius (blue). Figure 2‐IV‐B right showed the calculation of ZP thickness and perivitelline space. (V) Examples of time series of the morphokinetic parameters: Figure 2‐V‐A, B, C and D were the examples of the original and segmented time‐lapse images from a zygote. Figure 2‐V‐A represented the beginning frame captured right after fertilization check. Figure 2‐V‐B was the middle frame between Figure 2‐V‐A and Figure 2‐V‐D. Figure 2‐V‐C was the beginning of PN fusion. Figure 2‐V‐D was the frame right after PN fading. Figure 2‐V‐E, F, G and H gave examples of time series data of the zygote morphokinetic parameters. Figure 2‐V‐E was the cm_size, and it had a significant trend of decrease from the beginning to the end. Figure 2‐V‐F was the pn_size_mean. It was static before fusion, and its value sharply decreased when fusion began. Figure 2‐V‐G was the zp_thickness. Though it fluctuated, it did not have a significant trend. Figure 2‐V‐H was the zp_g_mean. Similar to Figure 2‐V‐G, though the value fluctuated, it was no trend on it. Both these two parameters were stationary

The results of 5‐fold cross‐validation in of total precision of cytoplasm segmentation were shown in Table 2. In each trial, on average, 974.4 images were used for training, while 243.6 images were used for testing. Accuracy was measured by the IoU. The proposed system showed high precision in cytoplasm segmentation. In each trial, IoU value was greater than 96.20% in both training and test phase. The average IoU of the proposed system achieved up to 97.01 ±1.33% in test phase and 97.23±1.43% in the training phase. There was no statistic significant between the training phase and test phase in each trial as well as between trials. Examples on the segmentation of cytoplasm were shown in Figure 2.

**TABLE 2:** The segmentation accuracy of day‐one human embryo cytoplasm, PN and ZP with 5‐fold cross‐validation

Structure	Trial	Training instances	Training accuracy (%)	Test instances	Test accuracy (%)
Cytoplasm	1	933	97.28 ± 1.26	285	97.21 ± 1.12
	2	987	97.06 ± 1.37	231	96.64 ± 1.34
	3	975	97.09 ± 1.45	243	97.34 ± 1.56
	4	995	97.47 ± 1.67	223	96.28 ± 1.78
	5	982	97.24 ± 1.89	236	97.57 ± 1.34
	Average	974.4	97.23 ± 1.43	243.6	97.01 ± 1.33
PN	1	586	91.35 ± 8.96	96	90.30 ± 8.25
	2	543	85.68 ± 8.45	139	83.74 ± 7.30
	3	461	82.73 ± 10.57	221	81.05 ± 7.19
	4	542	80.44 ± 7.38	140	81.83 ± 10.61
	5	596	87.22 ± 8.50	86	83.50 ± 9.35
	Average	545.6	85.45 ± 8.77	136.4	84.08 ± 11.34
ZP	1	235	81.83 ± 6.09	173	80.24 ± 5.17
	2	342	82.96 ± 5.62	66	75.80 ± 5.91
	3	335	81.13 ± 6.23	73	82.29 ± 5.17
	4	342	79.98 ± 5.59	66	82.97 ± 5.38
	5	378	79.74 ± 5.95	30	76.71 ± 6.91
	Average	326.4	81.12 ± 5.61	81.6	79.62 ± 7.34

Note

Instances of trainings and tests were recorded in the 5‐fold cross‐validation. Accuracy was measured by the IoU. Additionally, the difference of IoUs between the training phase and test phase in each trial as well as between trials were analysed with the Student *t* test.

There was no statistically significant difference between the training phase and test phase in each trial as well as between trials.

#### 3.1.2 PN segmentation

As the training images were of 2PN, we defined the left upper PN as PN1 and the other one as PN2. In most cases, the precision of PN segmentation was averaged over the IoU of both PN1 and PN2. However, in some frames, the pronuclei were fusing, and the inner boundaries of them were blurred (Figure 1, 2‐B). We considered the fusing PN as one object, and the IoU has counted the merged outline of labelled and recognized.

The results of 5‐fold cross‐validation in of total precision of PN segmentation were shown in Table 2. In each trial, on average, 545.6 images were used for training, while 136.4 images were used for testing. Accuracy was measured by the IoU. The proposed system showed high precision in PN segmentation. In each trial, IoU value was greater than 80.00% in both training and test phase. The average IoU of the proposed system achieved up to 90.30 ±8.25% in test phase and 91.35±8.96% in the training phase. There was no statistical significance between the training phase and test phase in each trial as well as between trials. Examples on the segmentation of PN were shown in Figure 2‐II.

#### 3.1.3 ZP segmentation

The results of 5‐fold cross‐validation in of total precision of ZP segmentation were shown in Table 2. In each trial, on average, 326.4 images were used for training, while 81.6 images were used for testing. Accuracy was measured by the IoU. The proposed system showed high precision in ZP segmentation. In each trial, IoU value was around 80.00% in both training and test phase. The average IoU of the proposed system achieved up to around 83% in both test and training phases. There was no statistically significant difference between the training phase and test phase in each trial as well as between trials. Examples on the segmentation of ZP were shown in Figure 2‐III.

### 3.2 Segmented values from the morphokinetic parameters

Because we used the images from the time‐lapse incubator, for morphometry, it had a series of values with time in a sample. These values were the dynamicity of the morphometry, which were so‐called ‘morphokinetic’. The morphometrics, with its morphokinetic values measured in a day‐one embryo, were defined as zygote (day‐one human embryo) morphokinetic parameters (ZMP). The ZMP discussed were listed in Table 3.

**TABLE 3:** The descriptions, ADF test results and values of the morphokinetic parameters

Structure	Morphokinetic parameters	Description	Selection based	Number of samples followed stationary hypothesis (%)	Stationary or non‐stationary parameter	Value of the parameters (n=24)^c^
Cytoplasm	cm_size	Size of cytoplasm	(Zhao*, et al*., 2019)^40^	0/24(0)	Non‐stationary	10073.35 ± 689.19 μm^2c^
	cm_r_mean	Mean of cytoplasm radius	Derivation	0/24(0)	Non‐stationary	56.37 ± 1.93 μm^c^
	cm_r_std	Deviation of cytoplasm radius	Derivation	0/24(0)	Non‐stationary	1.45 (0.99‐2.3)
	cm_i_mean	Mean of cytoplasm greyscale	(Scott, 2003)^41^	0/24(0)	Non‐stationary	119.37 (108.88‐132.87)
	cm_i_std	Deviation of cytoplasm greyscale	Derivation	0/24(0)	Non‐stationary	40.69 (37.86‐43.11)
ZP	zp_thickness	75th and above of ZP thickness	(Rienzi, Vajta, &Ubaldi, 2011)^10^	24/24(100)	Stationary	16.92 (15.75‐18.05) μm
	zp_thickness_std	Deviation of ZP thickness	Derivation	23/24(95.83)^b^	Stationary	3.36 (2.13‐4.43)
	zp_g_mean	Mean of ZP greyscale	(Sauerbrun‐Cutler*, et al*., 2015)^42^	23/24(95.83)^b^	Stationary	137.04 (121.69‐154.37)
	zp_g_std	Deviation of ZP greyscale	Derivation	23/24(95.83)^b^	Stationary	30.54 (25.77‐35.26)
	pvs_mean	Mean of perivitelline space	(Rienzi*, et al*., 2008)^43^	1/24(4.17)^b^	Non‐stationary	2.7 (1.96‐3.64) μm
PN	pn1_sz^a^	Mean value of PN size	(Scott, 2003)^41^	0/24(0)^d^	Non‐stationary^d^	503.19 (434.38‐557.54) μm^2d^
	pn2_sz^a^	Mean value of PN size	(Scott, 2003)^41^			
	pn _dist^a^	Distance of pn1 to pn2	(Beuchat*, et al*., 2008)^13^	0/24(0)	Non‐stationary	21.3 (18.59‐23.78) μm
	pn1_dist_cen^a^	The distance of pn1 to the geometrical centroid of cytoplasm	Derivation	0/24(0)	Non‐stationary	9.25 (7.32‐10.87) μm
	pn2_dist_cen^a^	The distance of pn2 to the geometrical centroid of cytoplasm	Derivation	0/24(0)	Non‐stationary	14.24 (12.5‐16.85) μm
	pn_fading	The duration from fertilization to PN disappearance	(Kaser and Racowsky, 2014)^44^			

Note

We selected morphokinetic parameters of cytoplasm, ZP and PN. The selections were based on the literature and on our previous studies. Stationary tests were conducted and parameters with the proportion higher than 70% following the hypothesis were considered as non‐stationary parameters. The descriptions and values of the parameters were given in the table as well.

^a^ The pn1 was defined as the PN that closer to the centroid of the cytoplasm.

^b^ The portion of accepting the stationary hypothesis is larger than 30%.

^c^ Normal distribution.

^d^ pn_size_med.

#### 3.2.1 Stationary test

For a day‐one embryo, it had lots of images captured at different time. Our CNN system segmented all its images, and the embryo had sequent values from the segmentation for different morphokinetic parameters. They were time series data, which is ‘an ordered sequence of values of a variable at equally spaced time intervals’ (Figure 2‐IV).^33^

For the stationary parameters, the value of each embryo was represented with the median of its time series value, which was a continuous variable. For the whole values of a specific parameter (eg zp_thickness), the Shapiro‐Wilk test was used to test its normality. Normally distributed continuous variables were described as mean and standard deviation, and for those not following a normal distribution, they were described as medians and interquartile ranges.

For the non‐stationary time series, the common practice of these data was to predict their future trends based on their fluctuation.^34^ However, in our study, the time series data of ZMP served as a part of the ‘fingerprint’ for themselves. There is no simple descriptive analysis for such time series data. We used the value of the median frame to represent the value of a non‐stationary parameter of a day‐one embryo.

All the samples of the fourteen morphokinetic parameters were examined by the ADF test to figure out the stationarity. The number of the portion rejected the stationary was lower than 5% in the cytoplasm and PN related parameters, which indicated they were non‐stationary parameters. Most of the ZP related parameters were higher than 95%, except the pvs_mean (Table 3). Examples of the time series curve of the morphokinetic parameters were shown in Figure 2‐V.

#### 3.2.2 Descriptive analysis for stationary parameters

Morphology metrics of the day‐one embryo structures were summarized into several parameters that were shown in Table 3. The stationary parameters were zp_thickness, zp_thickness_std, zp_g_mean and zp_g_std. For the stationary parameters, the value of each day‐one embryo was represented with the median of its time series value, which was a continuous variable. All of them were not normally distributed. The median and interquartile range (IQR) of these parameters were showed in Table 3. The zp_thickness was the value of length. The other parameters were dimensionless.

#### 3.2.3 Descriptive analysis for non‐stationary parameters

From Table 3, the non‐stationary parameters were cm_size, cm_r_mean, cm_r_std, cm_i_mean, cm_i_std, pvs_mean, pn_size_med, pn_dist, pn1_dist_cen and pn2_dist_cen. We used the value of the median frame to represent the value of a non‐stationary parameter of a day‐one embryo. The cm_size and cm_r_mean were normally distributed. The value of them was shown in mean±standard deviation. The median and IQR were shown in other parameters (Table 3). The cm_size and the pn_size_med were values of area while the cm_r_mean, pvs_mean, pn_dist, pn1_dist_cen and pn2_dist_cen were values of length. The other parameters were dimensionless.

#### 3.2.4 Comparison with other studies

With the descriptive data, we were able to compare with the values of morphometrics reported by other studies. The comparisons were shown in Table 4.

**TABLE 4:** Comparison with other studies

Structure	Morphokinetic parameters	Our data	Data from other studies	Resources
Cytoplasm	cm_size	10073.35 ± 689.19 μm^2^	9678±1245 μm^2^	Diéguez*, et al*.^35^
	cm_r_mean	56.37±1.93 μm	Max: 59±4 μm	
			Min: 53±7 μm	
ZP	zp_thickness	16.92 (15.75‐18.05) μm	16.6±3.2 μm	Bertrand*, et al*.^36^
			17.7±2.5 μm	Høst, *et al*.^37^
PN	pn_size_med	503.19 (434.38‐557.54) μm^2^	Diameter: Large: 25.2 ± 3.6 µm	Manor*, et al*.^38^
			Diameter: Small: 18.4 ± 3.9 µm	
			Diameter: Male: 16.5 ± 2.7 to 24.1 ± 2.2 µm	Payne*, et al*.^39^
			Diameter: Female: 15.3 ± 2.5 to 22.4 ± 2.3 µm	

Note

With the descriptive data of the morphokinetic parameters, the comparison of the values of morphometrics with published literature was conducted. We extracted data of the same parameters from other studies to compared with the data from CNN system to further confirm the accuracy and present reproducibility.

For the cytoplasm, the median data of time series was 10073.35 ± 689.19 μm^2^, and the radius was 56.37 ± 1.93 μm. The morphometrics of the size of zygote reported by Dieguez *et al*. were 9678 ± 1245 μm^2^ with a maximum radius of 59 ± 4 μm and minimum diameter of 53 ± 7 μm.^35^ Our data were comparable, which supported the precision of our segmentation algorithm. Moreover, the standard deviation of our data was smaller than Dieguez’s study, and it may be due to the more precision measurement method.

For the ZP, our data showed that the IQR of thickness was 16.92 (15.75‐18.05) μm. The Bertrand’s team reported the ZP thickness of fertilized oocytes was 16.6±3.2μm, and Høst’s team reported the ZP thickness of fertilized oocyte was 17.7 ± 2.5 μm.^36^, ^37^ Although our distribution of ZP thickness was different from these two teams (non‐normal distribution vs normal distribution), the range value of our data was comparable with their results. It should be noted that both the methods used in Bertrand’s team and Høst’ team were measurement of limited times with naked eyes by the embryologists, and our method used the whole ZP information.

For the PN size, our data was 503.19 (434.38‐557.54) μm^2^. Though the PN size changed with time, this result was the median of the PN time series, and it could represent the size of the PN in most of its time. The IQR range of our PN size data was comparable with those reported in Manor’s team and Payne’s team.^38^, ^39^

### 3.3 Elapsed time of the labelling/segmentation

During the manual segmentation by embryologists, it cost averagely 200s for each cytoplasm labelling, 150s for each PN labelling, and 300s for each ZP labelling. The time consumed to label the images of cytoplasm, PN and ZP were 243,600s, 102,300s and 122,400s, respectively. Totally, elapsed time of the manual labelling was 468,300s.

As to the automatic segmentation by CNN, the output of the segmentation of the cytoplasm, PN and ZP was produced parallelly. The elapsed time of the segmentation was 12.18s. Comparisons of the time consumed by the two methods were summarized as Table 5.

**TABLE 5:** Comparisons of the time consumed by the two methods

Structure	Operation	Manual operation	CNN (1 trial)
Cytoplasm	Number of images	1218	1218
	Average time/image	200s	0.01s
	Total time	243,600s	12.18s
PN	Number of images	682	682
	Average time/image	150s	0.01s
	Total time	102,300s	6.82s
ZP	Number of images	408	408
	Average time/image	300s	0.01s
	Total time	122,400s	4.08s
Elapsed time of the labelling/segmentation		468,300s	12.18s^a^

Note

We recorded the numbers of images and average time consumed of both of the manual labelling and CNN segmentation in different structures. The labelling of the three structures (in total 2308 images) was conducted one by one, while the CNN segmentation was conducted parallelly, which could obtain the results of three structures at a short time.

^a^ Segmented images of the three structures were produced parallelly.

## 4 DISCUSSION

In this study, we first presented the CNN algorithm for day‐one human embryo segmentation and tested its performance side‐by‐side with manual labelling. We then measured the segmented ZMP data and compared them with those reported by other studies to validate the precision of the segmentation of our CNN algorithm. Our CNN system has three distinctive advancements—high precision, high reproducibility and high speed.

One of the advancements of our CNN is high precision, embodies in the high accuracy of the segmentation of all the structures when compared side‐by‐side with the manual labelling. We are the first group invented and tested the CNN system on segmentation the full structures of the day‐one human embryo in time‐lapse images. We applied the system in segmenting the cytoplasm, PN and ZP of human time‐lapse images. For the human time‐lapse images, the precisions of the segmentation were that cytoplasm over 97%, PN over 84% and ZP around 80%.

The difficulties in ZP and PN segmentation should be noted. There were few studies on the segmentation on ZP and PN.^36^, ^37^, ^38^, ^39^ Previous studies only focused on the morphology images of single time points with naked eyes. It was hard to compare due to subjectivity that whether the precision of ZP and PN was high enough. For ZP segmentation, not all the embryos were in the centre of the embryo culture well, and in routine practice, more than half of them were located close to the edge of the well, in which parts of the ZP were blocked by the shadow of the well edge. This situation made embryologists difficult to label the whole part of the ZP, and for the blocked part of the ZP, our embryologists could only label by their experience, which was more subjective. For PN segmentation, the PN was small compared with the cytoplasm and the pixels labelled by the embryologists may not provide enough information to reveal the intrinsic morphology patterns. Also, the overlap of male and female PN made it difficult to distinguish the complete PN edge for each other, which compromised the quality of labelling.

Despite of the difficulties, the rest of the ZP (occupied most of the proportion) and PN were segmented correctly. Moreover, when calculating the ZP parameters, we could choose the maximum, minimum or mode to eliminate the impact of the blocked ZP. Considering the blocked and overlapped parts were labelled subjectively, the precision should be higher. Nevertheless, we were satisfied with this precision values of ZP and PN.

The second advancement is high reproducibility, the system enables automatic recognition in the comparable parameters as those in other studies. In order to demonstrate whether the precisions were enough to figure out the corresponding morphometrics for further application, we calculated the actual morphometrics of cytoplasm, PN and ZP and compared them with the reported results. For the morphometrics data of cytoplasm, ZP and PN, results such as cytoplasm size, thickness of ZP and PN size were comparable with those reported in other studies.^35^, ^36^, ^37^, ^38^, ^39^ From the comparison with the descriptive data reported by other studies, we further confirmed the precision of the segmentation algorithm.

The morphometrics data of the three structures calculated are the new potential morphometrics that measured by our novel segmentation algorithm. Because they were of kinetic – so‐called time series data, the traditional method for statistical analysis was not suitable for some of them. Therefore, we found out the stationary of these parameters first. For the stationary parameters, we treated them as tradition value by representing their time series with the median value. For the non‐stationary parameters, we choose the median value of their time series data as a sectional screenshot for representative, though it lost the kinetic information. With the descriptive data, we were able to compare with the morphometrics value reported by other studies. Therefore, we have more confidence in the biological information provided by those derivation parameters that have not been discussed before.

The third advancement is high speed, capable to label in the short‐elapsed time of the segmentation time compared with the laborious manual labelling. In total, there were 1218, 682 and 408 images for cytoplasm, PN and ZP segmentation respectively. It cost more than 130 hours to finish the manual labelling. In routine IVF practice, it is impossible for embryologist to spend lots of time in labelling the embryos for further morphological analysis. Nevertheless, in our study the CNN system only needed 12.18 seconds to finish the segmentation, which makes the further morphological analysis on day‐one embryo is ready for routine practice.

The process of analysing the plentiful images of embryos generated by TLI is toilsome. Some minor but important changes cannot be recognized by naked eyes. The automatic process of the CNN segmentation saved much time for the embryologists to analyse the day‐one embryos. Our system would be a helpful tool to reduce the workload to a great extent compared with manual labelling and improved the work efficiency.

Additionally, our CNN system did not incur any adverse consequences on embryo; it is a novel non‐invasive procedure that can be applied anytime, anywhere without any limitation in IVF clinics equipped with the time‐lapse incubator. We can analyse the features of the day‐one human embryo that could not be usually perceived quantitatively by the naked human eye. At the same time, the inter‐observer and intra‐observer variations can be prevented with the automatic process of CNN system, which contribute to a more objective result.

In future, we will establish a cloud service platform, test its performance and run the prediction model on it with more collaborated IVF centres. During the test, we will collect more results of embryo development as well as image data, which will be used to improve our CNN segmentation system and get more precise ZMP. It may also provide a considerable potential for the selection of embryos and prediction of the embryo development, making the process faster, easier and more accurate with three distinctive advancements. In conclusion, our CNN system is ready to be applied in practice for day‐one human embryo segmentation as a robust tool with high precision, reproducibility and speed.

## CONFLICT OF INTEREST

All authors approved this submission and declare no potential conflicts of interest.

## AUTHOR CONTRIBUTIONS

Mingpeng Zhao: Writing‐original draft (equal); data curation (equal); formal analysis (equal); methodology (equal); investigation (equal). Murong Xu: Writing‐original draft (equal); data curation (equal); formal analysis (equal); investigation (equal). Hanhui Li: Methodology (equal). Odai Alqawasmeh: Methodology (equal). Jacqueline Pui Wah Chung: Resources (equal). Tin Chiu Li: Supervision (equal); resources (equal). Tin‐Lap Lee: Supervision (equal). Patrick Ming‐Kuen Tang: Project administration (equal); supervision (equal); Writing‐review and editing (equal); David, Yiu Leung Chan: Resources (equal); funding acquisition; project administration (equal); supervision (equal); writing‐review and editing (equal).

## Supporting information

Fig S1

Fig S1_1
