# Supplementary material for: Application of convolutional neural network on early human embryo segmentation during in vitro fertilization
Source: J Cell Mol Med. 2021 Jan 24;25(5):2633–44. doi: 10.1111/jcmm.16288 (PMC7933952; doi:10.1111/jcmm.16288)
Supplement: Supplementary file 1 — Fig S1 [file JCMM-25-2633-s001.docx]

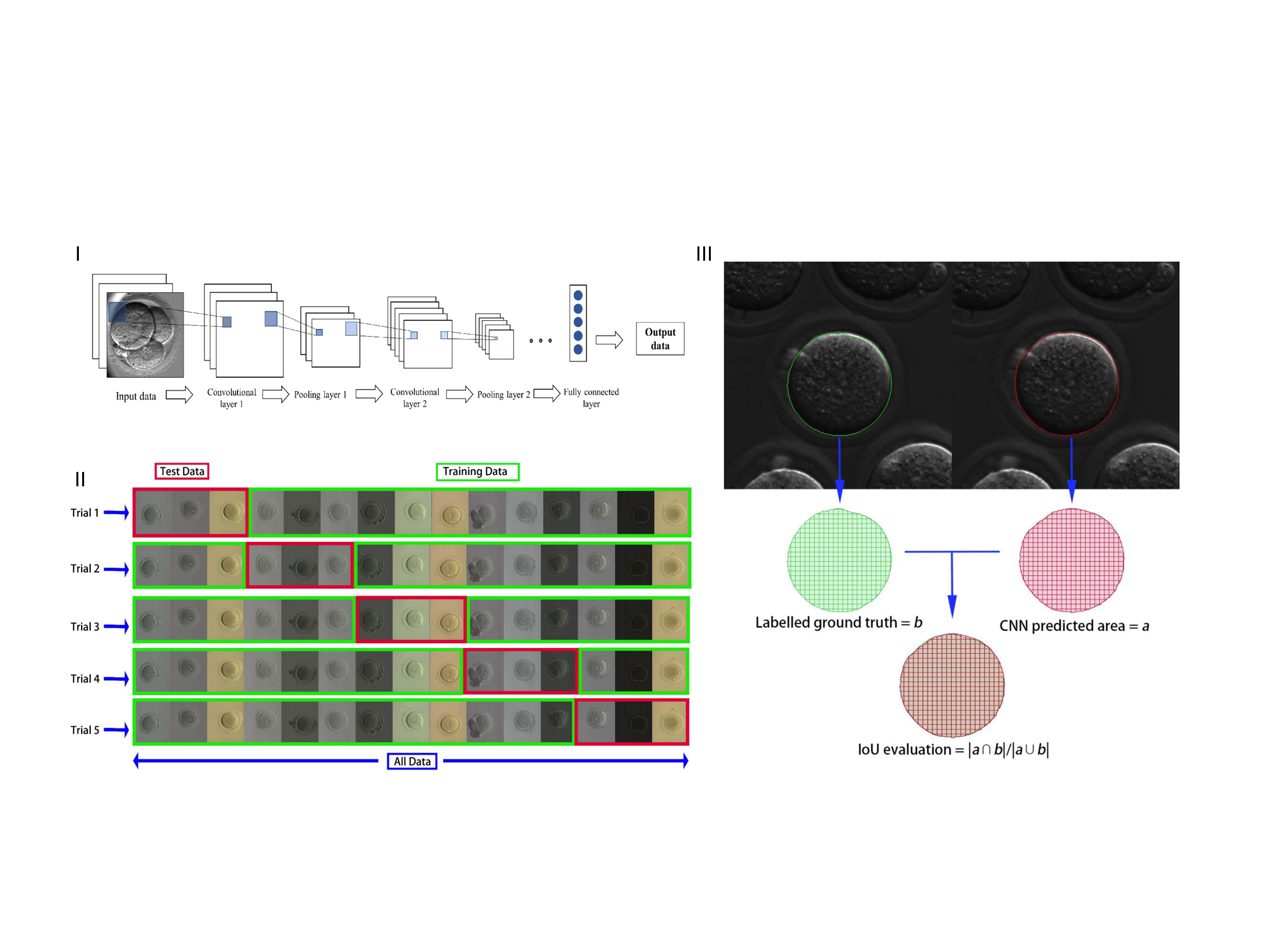


**Supplement-1**

**(I) Schematic of CNN:** A typical CNN consists of the layers of convolutional, pooling, and fully connected. During the convolution process, filter kernels scan the input image to form feature maps. Then the maps are processed by a pooling layer. After several times of pooling & convolutional, the output data is generated in the fully connected layer.

**(II) Illustration of cross-validation:** The dataset was evenly divided into five parts randomly. In each cross-validation trial, one part was designed as test data (red) the rest performed as training data (green). After one trial, one of the rest four parts were chosen as test data while others were as training data. This process finished when all five parts were tested.

**(III) Illustration of IoU:** The green circle represented the area labelled by embryologist while the red circle represented for the area recognized by the system. The intersection meant the common area in both green and red circle while the union means the whole area in the combined circle.
